# The global burden and risk factors of cardiovascular diseases in adolescent and young adults, 1990–2019

**DOI:** 10.1186/s12889-024-18445-6

**Published:** 2024-04-12

**Authors:** Zhuang Tong, Yingying Xie, Kaixiang Li, Ruixia Yuan, Liang Zhang

**Affiliations:** 1 Clinical Big Data Center, The First Affiliated Hospital of Zhengzhou University, Zhengzhou, Henan Province China; 2 Henan Academy of Medical Big Data, Zhengzhou, China; 3 Department of Scientific Management, The First Affiliated Hospital of Zhengzhou University, Zhengzhou, Henan Province China; 4 Department of Cardiovascular Surgery, Rhe First Affiliated Hospital of Zhengzhou University, Zhengzhou, Henan Province China

**Keywords:** Cardiovascular disease, Adolescent and young adults, Disease burden, Risk factors

## Abstract

**Background:**

To provide details of the burden and the trend of the cardiovascular disease (CVD) and its risk factors in adolescent and young adults.

**Methods:**

Age-standardized rates (ASRs) of incidence, mortality and Disability-Adjusted Life Years (DALYs) were used to describe the burden of CVD in adolescents and young adults. Estimated Annual Percentage Changes (EAPCs) of ASRs were used to describe the trend from 1990 to 2019. Risk factors were calculated by Population Attributable Fractions (PAFs).

**Results:**

In 2019, the age-standardized incidence rate (ASIR), age-standardized mortality rate (ASMR) and age-standardized DALYs rate (ASDR) of CVD were 129.85 per 100 000 (95% Confidence interval (CI): 102.60, 160.31), 15.12 per 100 000 (95% CI: 13.89, 16.48) and 990.64 per 100 000 (95% CI: 911.06, 1076.46). The highest ASRs were seen in low sociodemographic index (SDI) and low-middle SDI regions. The burden was heavier in male and individuals aged 35–39. From 1990 to 2019, 72 (35.29%) countries showed an increasing trend of ASIR and more than 80% countries showed a downward trend in ASMR and ASDR. Rheumatic heart disease had the highest ASIR and Ischemic Heart Disease was the highest in both ASMR and ASDR. The main attributable risk factor for death and DALYs were high systolic blood pressure, high body-mass index and high LDL cholesterol.

**Conclusions:**

The burden of CVD in adolescent and young adults is a significant global health challenge. It is crucial to take into account the disparities in SDI levels among countries, gender and age characteristics of the population, primary types of CVD, and the attributable risk factors when formulating and implementing prevention strategies.

**Supplementary Information:**

The online version contains supplementary material available at 10.1186/s12889-024-18445-6.

## Introduction

Cardiovascular disease (CVD) is a major threat to human health [1]. At different ages of life, individuals may be at risk of CVD. Atherosclerosis beginning in childhood is the pathological basis of CVD [2, 3]. In addition, although the hard endpoints of CVD such as stroke or heart attack usually do not occur in childhood, evidence shows that the manifestation of CVD risk factors begins in childhood, especially poor diet and obesity [4]. For children and young adults, rheumatic heart disease (RHD) is the leading cause of heart failure and the most common type of CVD requiring cardiac surgery, especially in low- and middle-income countries [5]. After adulthood, the role of age as an independent risk factor for CVD becomes more obvious [6], and CVD is the primary type of disease that plunders life and affects quality of life until old age.

Adolescents and young adults are usually described as a subgroup between children and middle-aged and elderly people, and 15–39 years old is widely recognized as the age division of this group [7]. This period of time serves as a link between past and present in our lives [8] and this age group may also bear the burden of physical and psychological changes. However, adolescent and young adults does not receive the same special attention to meet the disease onset type and age-related treatment and care needs as children or older adults in existing healthcare services. Studies have reported the global burden and trends of CVD [9]. To the best of our knowledge, a comprehensive assessment of the CVD burden in the adolescent and young adults is largely unknown or unreported.

The Global Burden of Disease 2019 (GBD 2019) study released death and health losses from 369 diseases, injuries and impairments and 87 risk factors in 204 countries and regions around the world from 1990 to 2019 [10]. Therefore, it provides an excellent opportunity to systematically analyze CVD disease burden and risk factors of adolescent and young adults globally and in different countries. In this study, we aimed to analyze and report CVD burden estimates in this distinctive population, using age standardized rates and EAPC to focus on comparing differences of this burden distribution and changes by gender, and regions with different levels of economic development, and to describe risk factors using attributable risk.

## Methods

### Data source

GBD 2019 was a multinational collaborative study that estimated the burden of disease in every country in the world. Underlying data sources were censuses, household surveys, civil registration and vital statistics, disease registries, disease notification, use of health services, air pollution monitors, satellite imaging and other sources [10, 11]. Through systematic comprehensive evaluation and standardization, the GBD 2019 estimated the incidence, mortality and disability adjusted life years (DALYs) associated with diseases and injuries. More details of the calculation methods had been revealed in previous publications [10, 12]. According to the purpose of this study, we extracted data on incidence, mortality, DALYs and risk factors of CVD in the age range of 15–39 years in GBD 2019.

### Estimation of cardiovascular diseases burden in GBD 2019

GBD 2019 divides all causes into four levels. Cardiovascular diseases was classified as the second level, belonging to the first level of non-communicable diseases, and are further divided into 11 types at the third level, which including Rheumatic heart disease, Ischemic heart disease, Stroke, Hypertensive heart disease, Cardiomyopathy and myocarditis, Atrial fibrillation and flutter, Aortic aneurysm, Peripheral artery disease, Endocarditis, Non-rheumatic valvular heart disease, Other cardiovascular and circulatory diseases. Each of them identified with standard case definitions [9]. 

Mortality was calculated as number of deaths (n)×100,000/population and Cause of Death Ensemble model and spatiotemporal Gaussian process regression were used to calculate cause-specific death rates [12, 13]. Incidence rate was calculated as number of new cases (n) ×100,000/population and DALYs was the sum of years of life lost (YLLs) and years lived with disability (YLDs) [14]. GBD 2019 used the Bayesian meta-regression modeling tool DisMod-MR2.1 to ensure consistency between these outcome indicators of disease burden. Uncertainty intervals (UIs) were provided for every point estimate using the 25th and 75th ordered 1000 draw values of the posterior distribution [10].

### Estimation of attributable risk factors for CVD in GBD 2019

In GBD 2019, a rule-based synthesis of evidence to ensure comparable quantification of risk over time and across populations was used to calculate the disease burden attributable to risk factors [11]. The global disease burden of CVD in people aged 15–39 can be attributed to three categories of Behavioral risks, Environmental/occupational risks and Metabolic risks, with a total of 27 detailed risk factors. They included Ambient particulate matter pollution, Smoking, Alcohol use, High body-mass index, Diet low in fruits, Diet high in sodium, Kidney dysfunction and so on.

### Statistical analysis

The age-standardized incidence rate (ASIRs), age-standardized mortality rate (ASMR) and age-standardized DALYs rate (ASDR) with 95% UIs were used to describe the disease burden of CVD in adolescent and young adults. The following equation was used to calculate the age-standardized rates (ASRs): $\text{a}\text{g}\text{e}-\text{standardized rate = }\frac{{\sum }_{i=1}^{A}{a}_{i}{w}_{i}}{{\sum }_{i=1}^{A}{w}_{i}}$. Where a_i_ was the age-specific rate and w_i_ was the weight in the same age subgroup of the chosen reference standard population, which was the world standard population (WHO 2000–2025) [11].

The estimated annual percentage changes (EAPCs) in the ASRs were used to evaluate the trend in the burden of CVD in adolescent and young adults. The natural logarithm of the ASR was fitted to the following regression line model: ln (ASR) = α + βx + ɛ, where x is the calendar year. The EAPC and its 95% confidence interval (CI) were derived from the following regression model: y = 100×(exp (β) − 1), where y is the EAPC. When the 95%CI lower limit of the estimated EAPC was greater than 0, ASRs was considered to be on the rise during the observation period. On the contrary, when the upper limit of 95%CI of the EAPC estimate is less than 0, there is a downward trend. When 95%CI contains 0, the trend change is not statistically significant [15, 16]. The Population Attributable Fractions (PAFs) were used to compare the effects of different risk factors [17]. Data collation and analysis and graphical rendering were performed using Python and R software (version 3.5.3).

## Results

### The ASIR of CVD in individuals aged 15–39 years

**Fig. 1:**
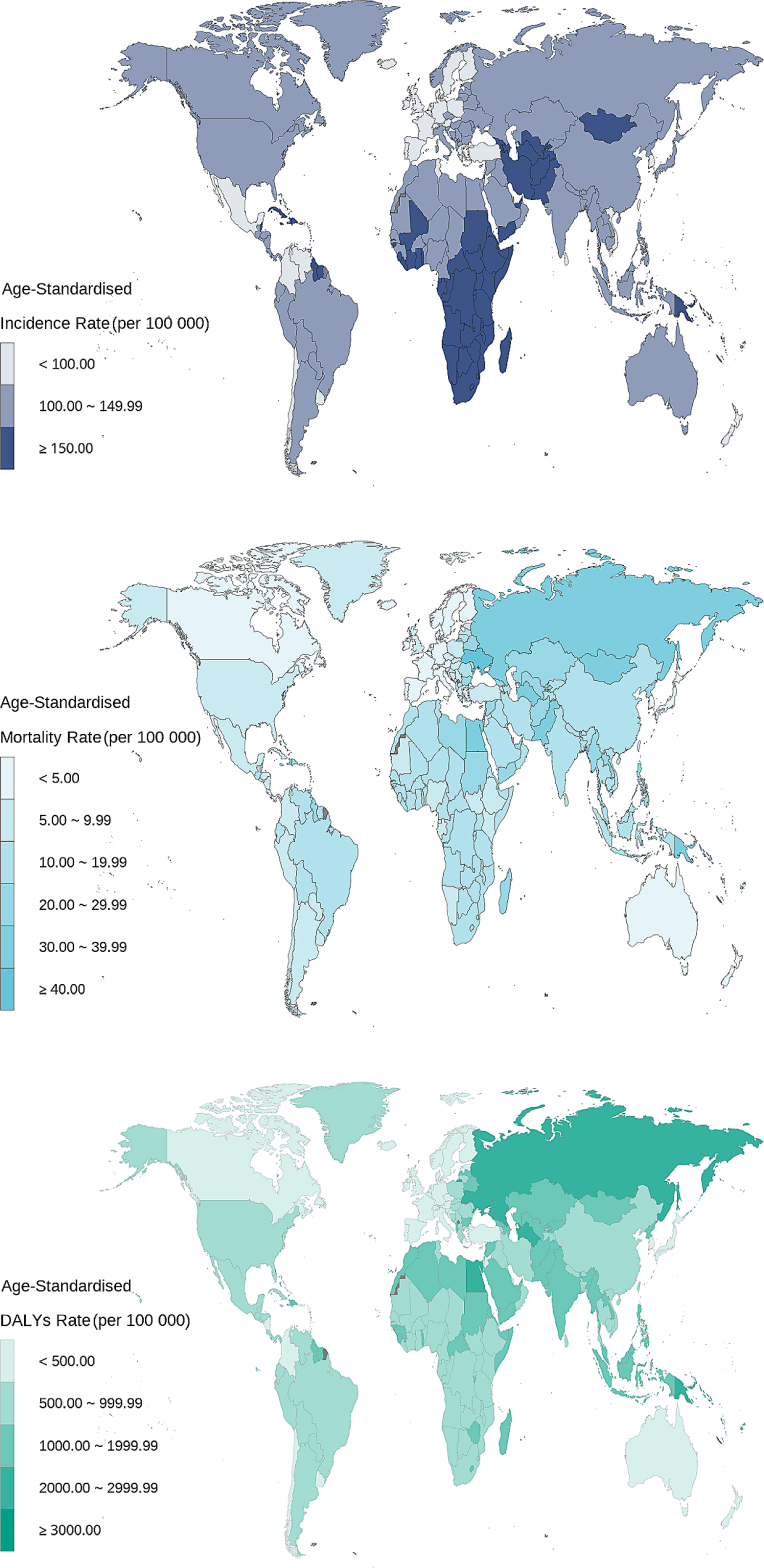
The distribution of cardiovascular disease (CVD) burden in adolescent and young adults by countries and regions in 2019

In 2019, there were an estimated 3.87 million (95% UI: 332.70-446.79) incident CVD cases among individuals aged 15–39 years worldwide and the ASIR was 129.85 per 100 000 (95% CI: 102.60, 160.31). The highest ASIRs were seen in low SDI and low-middle SDI. (Table 1). The countries with higher ASIR were mainly distributed in sub-Saharan Africa, Central Asia, Western Asia, and northern part of East Asia. (Fig. 1).

**Table 1:** The burden of CVD in 1990 and 2019 and the temporal trends from 1990 to 2019 in adolescent and young adults

Characteristics	Incidence, thousands (95% UI)	Age-standardised incidence rate per 100 000 (95% CI)	Mortality, thousands (95% UI)	Age-standardised mortality rate per 100 000 (95% CI)	DALYs, thousands (95% UI)	Age-standardised DALY rate per 100 000 (95% CI)
Global	3868.17(3327.04-4467.90)	129.85(102.60,160.31)	455.85(420.27-493.99)	15.12(13.89,16.48)	29781.21(27619.58-32143.30)	990.64(911.06,1076.46)
Female	1863.30(1593.93–2178.00)	126.88(98.94,157.80)	155.79(138.59-171.69)	10.46(9.25,11.63)	11093.48(9974.11-12271.52)	747.66(666.18,831.43)
Male	2004.87(1736.72-2302.27)	132.84(106.16,163.08)	300.06(274.75-327.34)	19.70(17.93,21.67)	18687.74(17204.98-20373.45)	1229.51(1120.67,1349.99)
SDI						
Low SDI	731.14(590.75-899.01)	164.40(121.74,147.68)	69.60(60.94–79.43)	17.49(15.17,20.19)	4741.71(4174.52-5364.88)	1158.85(1012.13,1327.23)
Low-middle SDI	1079.45(897.21-1278.33)	147.68(112.84,186.54)	140.59(126.72-156.57)	19.98(17.80,22.51)	9041.46(8209.26-9932.75)	1273.45(1139.06,1421.23)
Middle SDI	1131.99(978.83-1310.94)	119.64(94.56,147.97)	146.88(133.72-160.38)	15.17(13.70,16.66)	9603.89(8766.57-10459.33)	998.02(903.58,1092.93)
High-middle SDI	538.41(481.83-609.59)	98.05(81.39,118.04)	75.43(69.93–80.86)	12.88(11.90,13.88)	4774.72(4422.21-5115.35)	830.49(764.98,896.38)
High SDI	384.54(346.32-424.69)	107.96(92.71,125.15)	23.01(21.67–25.04)	6.22(5.79,6.80)	1596.98(1475.67-1738.99)	440.65(403.59,485.84)
Cardiovascular diseases						
Rheumatic heart disease	1477.52(999.63-1985.7)	50.37(28.88,74.15)	39.55(34.20-44.71)	1.32(1.11,1.53)	3457.74(2886.30-4129.60)	116.18(95.10,141.44)
Ischemic heart disease	795.57(604.16-1024.99)	26.33(17.15,37.94)	212.22(194.81-233.58)	7.02(6.41,7.76)	12153.73(11176.20-13343.37)	402.58(367.59,445.01)
Stroke	825.67(677.52-1027.35)	27.48(20.21,37.24)	126.38(115.81–138.5)	4.19(3.79,4.62)	9133.13(8273.99-10048.86)	303.96(273.40,336.20)
Hypertensive heart disease	0	0	15.48(11.39–17.76)	0.51(0.37,0.59)	896.54(662.15-1029.11)	29.68(22.01,34.22)
Cardiomyopathy and myocarditis	331.24(229.90-448.34)	11.12(6.43,17.56)	27.56(22.43–30.47)	0.91(0.73,1.03)	1621.33(1329.5-1790.06)	53.87(43.11,60.64)
Atrial fibrillation and flutter	71.88(35.69-127.93)	2.36(1.17,4.21)	0.23(0.19–0.26)	0.007(0.006,0.008)	32.79(20.96–49.89)	1.08(0.68,1.65)
Aortic aneurysm	-	0	4.27(3.91–4.76)	0.14(0.13,0.16)	243.23(222.55-271.43)	8.07(7.33,9.02)
Peripheral artery disease	0	0	-	0	-	0
Endocarditis	171.15(115.04-240.99)	5.73(3.02,9.13)	6.83(5.61–8.01)	0.23(0.18,0.28)	404.46(334.01-472.41)	13.50(10.91,16.38)
Non-rheumatic valvular heart disease	195.15(179.89-212.37)	6.46(5.95,7.03)	4.41(3.72–5.22)	0.15(0.12,0.18)	263.91(222.37-313.54)	8.85(7.38,10.55)
Other cardiovascular and circulatory diseases	0	0	18.93(16.92–21.26)	0.63(0.56,0.72)	1574.34(1321.73–1915.00)	52.86(43.17,66.87)

In terms of trends, the change in ASIR attributable to CVD among people aged 15–39 years showed a nonsignificant global upward trend from 1990 to 2019, with an EAPC point estimate of 0.04 and a 95%CI containing zero. In different SDI regions, high-middle SDI regions and middle SDI regions showed regional downward trends, while other SDI regions remained stable. In all 204 countries, 72 (35.29%) countries showed an increasing trend of ASIR, mainly distributed in Western Asia, Southeast Asia, East Asia, North Africa and Eastern Europe. Especially in Saudi Arabia, EAPC of ASIR was 1.25 (95%CI: 1.21, 1.29). (Table 1; Fig. 1).

In different genders, from 1990 to 2019, ASIR attributable to CVD showed a slight upward trend in male aged 15–39 years, and the EAPC of ASIR was 0.09 (95%CI: 0.04, 0.14), while the trend in female was not statistically significant. Among different age groups, EAPCs of ASIRs were 0.52 (95%CI: 0.45, 0.60) and 0.11 (95%CI: 0.01, 0.21) in age group 15–19 and 20–24, respectively. There was no significant decrease in ASIR in age group 25–29, while the burden of CVD in other age groups showed a downward trend. (Figure S1).

### The ASMR and ASDR of CVD in individuals aged 15–39 years

In 2019, there were 455.85 thousand (95% UI: 420.27-493.99) deaths attributed to CVD among individuals aged 15–39 years and the DALYs were 29.78 million (95%UI: 27.62–32.14). The ASMR and ASDR were 15.12 per 100 000 (95% CI: 13.89, 16.48) and 990.64 per 100 000 (95% CI: 911.06, 1076.46). (Table 1). Countries with heavier burden of ASMR and ASDR were concentrated in Eastern Europe, North Asia, Central Asia, West Asia and North Africa. The highest ASMR was seen in Kiribati and it was nearly 41 times higher than that of the lowest country in Switzerland, which were 90.11 per 100 000 (95% CI: 70.64, 112.92) and 2.22 per 100 000 (95% CI: 1.96, 2.52), respectively. (Fig. 1).

Compared with female, this burden was heavier in male. The ASMRs of female and male were 19.70 per 100 000 (95% CI: 17.93, 21.67) and 10.46 per 100 000 (95% CI: 9.25, 11.63), respectively. Of all five age groups, individuals aged 35–39 years had the largest contribution, with corresponding rates of death and DALYs of 34.93 per 100 000 (95% UI:32.24–37.80) and 1976.74 per 100 000 (95% UI:1836.29-2125.87). (Table 1, Figure S1).

**Fig. 2:**
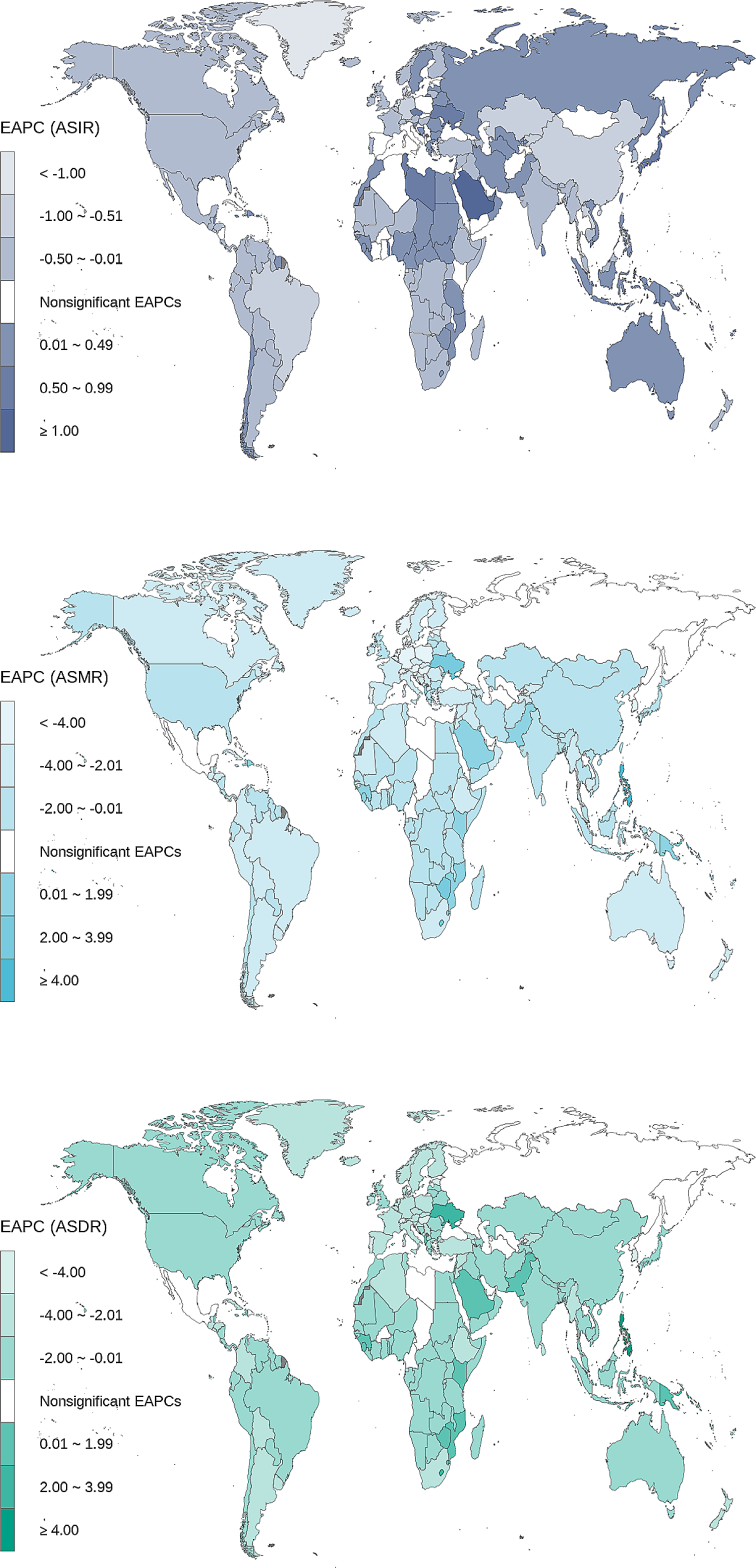
Temporal trend of cardiovascular disease (CVD) burden in adolescent and young adults by countries and regions from 1990 to 2019

The changes of ASMR and ASDR attributable to CVD among people aged 15–39 years showed global downward trends and the values of EAPC were 0.90 (95% CI: 1.01, 0.78) and 0.80 (95% CI: 0.90, 0.71), respectively. (Table 1). The ASMR and ASDR showed the most obvious regional downward trend in high SDI regions and high-middle SDI regions. The values of EAPC of ASMR were − 1.46 (95%CI: -1.58, -1.34) and − 1.25 (95%CI: -1.45, -1.05), and of ASDR were − 1.17 (95%CI: -1.28,-1.06) and − 1.15 (95%CI: -1.32, -0.98). (Fig. 2).

In all 204 countries, 167 (81.86%) countries showed a downward trend and 23 (11.27%) countries showed an upward trend, with the most obvious seen in the Philippines, where EAPC of ASMR was 5.80 (95%CI: 4.79, 6.81). The countries with an upward trend were mainly distributed in South Asia, Southeast Asia, East Asia, sub-Saharan Africa and Northern Europe. Similar to ASMR, the ASDR of most countries showed a downward trend, while 21 countries increased, and 16 countries kept stable. (Fig. 2).

**Fig. 3:**
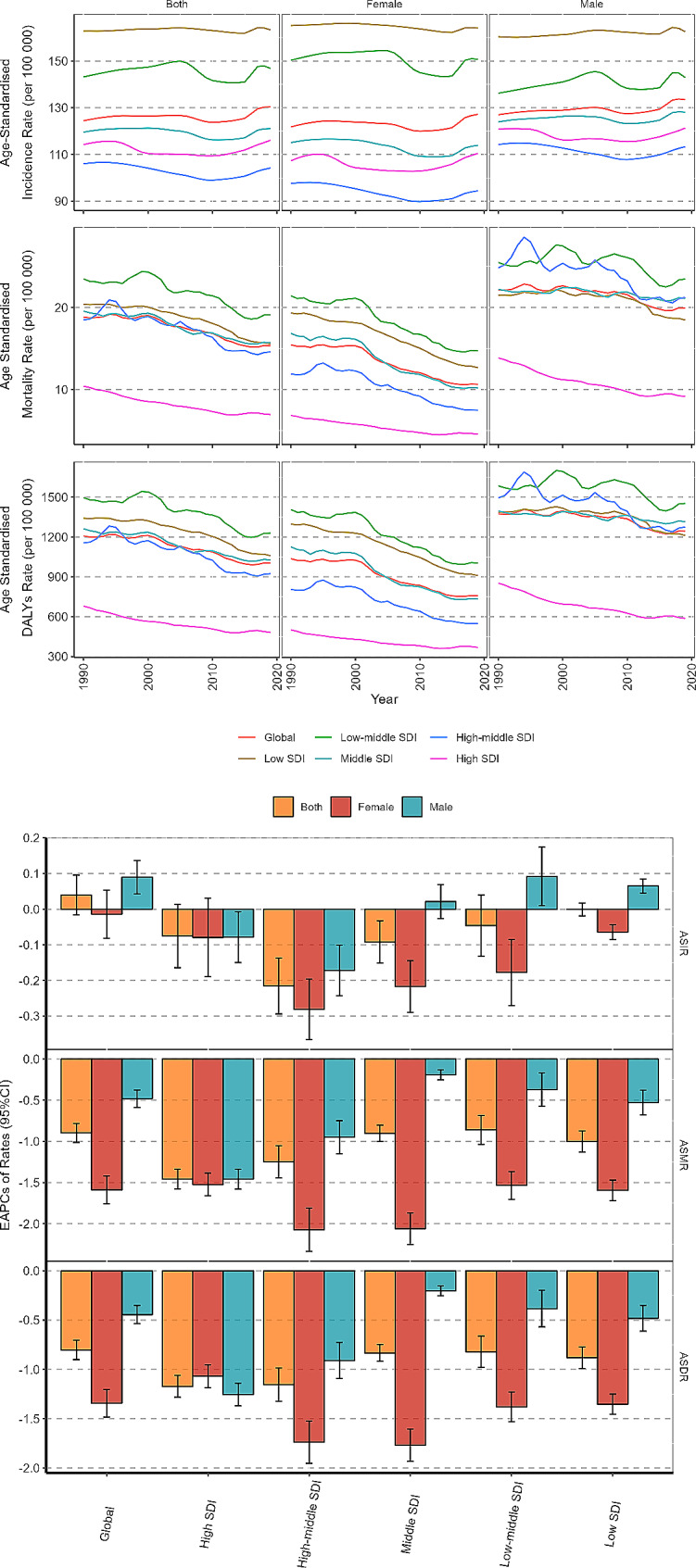
Temporal trend of cardiovascular disease (CVD) burden in adolescent and young adults by sex and SDI from 1990 to 2019

The ASMR and ASDR of different genders showed a downward trend, and it was more obvious in female, especially in the gap of ASMR decline. EAPCs of ASMR were − 1.59 (95%CI:-1.76,-1.42) in female and − 1.34 (95%CI:-1.48,-1.20) in male, EAPCs of ASDR were − 0.48 (95%CI: -0.59, -0.38) in female and − 0.44 (95%CI: -0.54, -0.35) in male. (Table 1; Fig. 3)

CVD types in individuals aged 15–39 years.

**Fig. 4:**
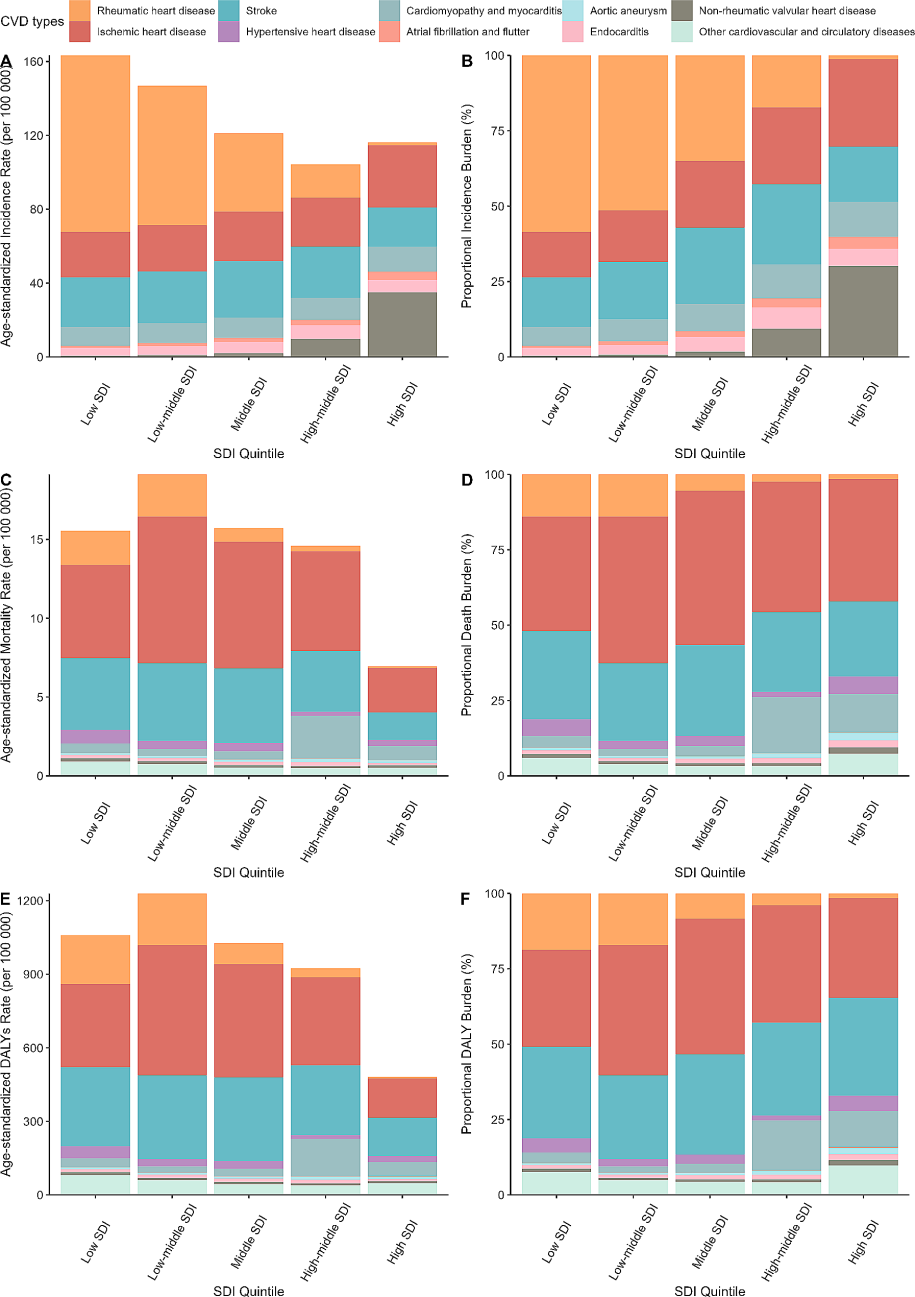
Proportion of Cardiovascular disease (CVD) types globally in adolescent and young adults by SDI in 2019

RHD had the highest ASIR in individuals aged 15–39 years, which was 50.37 per 100 000 (95%CI: 28.88, 74.15), followed by Stroke and Ischemic heart disease (IHD). IHD was the highest in both ASMR and ASDR, with corresponding rates of 7.02 per 100 000 (95%CI: 6.41, 7.76) and 402.58 per 100 000 (95%CI: 367.59, 445.01). RHD had the highest burden in 15–19 years old and there was a decline with age in individuals aged 15–39 years. The other several types were characterized by the opposite age distribution. In addition, the burden of RHD was the highest in low SDI and low-middle SDI. The ASIR of Non-rheumatic valvular heart was much higher in high SDI. (Fig. 4, Figure S2).

### Attributable risk factors for death and DALY of CVD in individuals aged 15–39 years

**Fig. 5:**
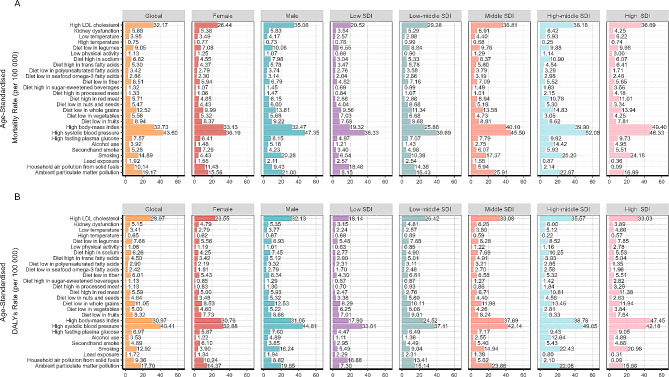
Proportion of cardiovascular disease (CVD) death and disability adjusted life years (DALYs) in adolescent and young adults attributable to 27 risk factors by sex and SDI in 2019

There were 27 detailed risk factors attributable to death and DALY in CVD in individuals aged 15–39 years in GBD 2019. High systolic blood pressure, high body-mass index and high LDL cholesterol were the top three risk factors. PAFs of ASMR were 43.60%, 32.73% and 32.17%, PAFs of ASDR were 40.41%, 30.97% and 28.97%, respectively. The PAFs for ambient particulate matter pollution, smoking, a diet low in whole grains, and household air pollution from solid fuels range from 9 to 19%. (Fig. 5).

The distribution had some gender and age characteristics. The PAFs of smoking for deaths and DALYs were 20.28% and 18.24% in male, which was nearly five times higher than that in female. Other risk factors such as ambient particulate matter pollution, diet low in whole grains, diet high in sodium and alcohol use also played a more obvious role in male. For female, secondhand smoke and household air pollution from solid fuels were the most typical influencing factors, which PAFs of ASMR were 7.29% and 11.48% and PAFs of ASDR were 6.10% and 10.24%, both higher than those of male. In different age groups, alcohol use and high systolic blood pressure were more obvious with the increase of age. The effect of temperature was more consistent in different age groups. (Fig. 5, Figure S3).

Compared with regions with a low SDI, death burden for early onset CVD in regions with a high SDI were more attributable to ambient particulate matter pollution (16.99% *v* 8.15%), smoking (24.18% *v* 6.54%), high body-mass index (49.40% *v* 19.32%), diet high in red meat (11.01% *v* 2.68%), diet high in processed meat (4.18% *v* 0.84%), diet high in sugar-sweetened beverages (3.56% *v* 0.69%), low physical activity (3.00% *v* 0.68%) and high LDL cholesterol (36.69% *v* 20.52%). In contrast, contributions to death were greater in regions with a low SDI for household air pollution from solid fuels (18.48% *v* 0.09%), lead exposure (2.57% v 0.36%) and diet low in vegetables (7.03% v 4.25%) compared with a high SDI. The attributable risk factors for DALYs were similar. (Fig. 5, Figure S3).

## Discussion

### Statement of principal findings

The disease burden of CVD in adolescents and young adults and its changing trend are important public health issues worthy of attention. In this study, we systematically analyzed the study data of GBD 2019 and there were some clear findings. In terms of absolute numbers, compared with other major chronic non-communicable diseases, such as cancer [8], the incidence, mortality and DALYs of CVD in adolescent and young adults are heavier. From the perspective of changing trends, the incidence rate of CVD in adolescents and young adults keep stable except in male, and the mortality and DALYs rate were decreasing. The distribution and changing trend of the burden have distinct characteristics related to the level of socioeconomic development, age and gender. We also found that the strength of the attributable risk factors for early onset CVD differed by age, gender, and super regions. These findings can provide clues to understanding the nature of the CVD epidemic in adolescent and young adults and call for action from a global perspective to address this public health problem.

Regional difference in the burden of CVD in adolescent and young adults.

Previous studies on early onset CVD mainly focused on the risk factors in adolescents and young adults [3, 18, 19]. Tomi T et al. [20] followed 856 Finnish participants with a mean age of 15 years for 21 years and suggested that the pursuit of ideal cardiovascular health in childhood had positive implications for the prevention of cardiometabolic outcomes in adulthood. It is critical to understand the CVD burden and regional distribution in this distinctive population worldwide. Based on the GBD 2019 data, we analyzed the data of different SDI levels and found that the age-standardized rate of incidence, death and DALYs of CVD among people aged 15–39 years were more serious in regions with lower SDI. This suggested that this distinctive population face a large burden of CVD disease globally and there are serious regional inequalities. In addition, it is worth pointing out that incidence, mortality rates, DALYs and DALY rates were higher in the low SDI regions than in the low-middle SDI regions, which may be attributed to poorer screening and diagnosis conditions [21, 22]. Therefore, there is an urgent need to take measures to control CVD transmission in adolescents and young adults, especially in countries with less socioeconomic development.

We found that RHD was the most prevalent type of CVD in population aged 15–39 years, and most of them occurred in countries or regions with low or low-middle SDI. Although reducing streptococcal infection by injection of antibiotics can prevent the occurrence of RHD [23], risk factors such as lack of antibiotics, overcrowded living conditions, and poor living and sanitary conditions are common in underdeveloped areas [24]. At the same time, the high incidence of Non-rheumatic valvular heart disease in adolescent and young adults in high SDI countries was worthy of attention. It is an independent disease type affecting health, and closely related to arrhythmia and atrial fibrillation [25]. In recent years, most reports on Non-rheumatic valvular heart disease focused on the elderly [26], and attention to adolescents and young adults is lacking. This suggests that we should pay full attention to the characteristics of CVD types under different levels of economic development when improving cardiovascular health services for adolescents in the future.

Sex and age differences in the burden of CVD in global adolescents and young adults.

When “inequity” is mentioned in the study of disease burden, it is not only the impact of socioeconomic development, but also gender and age stage should not be ignored [27, 28]. In this study, we found that gender and age were important factors influencing the differences in distribution and change of CVD burden among people aged 15 to 39 years. Compared with female, the burden and change trend of incidence, mortality and DALYs of CVD was heavier in male. Firstly, the reason may be that estrogen and the resulting HDL in premenopausal women are to a large extent cardioprotective [29], delaying the onset age of CVD. Secondly, established risk factors for CVD, such as tobacco, alcohol, unhealthy eating habits, and poor initiative to seek external support when stressed, are more common in males [30]. In addition, the increasing trend of CVD incidence in adolescents aged 15–19 years is of concern, especially in females.

### Risk factors in the burden of CVD in global adolescents and young adults

High systolic blood pressure is a major risk factor for CVD events and mortality [31]. Similar results were found in our analysis. HSBP is the leading cause of CVD burden in global adolescents and young adults with PAF over 40%. We also found important effects of high body-mass index and high LDL cholesterol on CVD burden, both with PAF exceeding 30%. A previous study [32] showed that a greater proportion of deaths from cardiovascular causes occurred in higher BMI categories in younger adults who are overweight and obese. The increasing prevalence of hypertension and obesity among children and adolescents has become important public health issues [33, 34]. In 2016, the global number of girls and boys who were obese was 50 million and 74 million respectively, while 213 million children and adolescents were overweight but below the obesity threshold [35]. This suggests that the control and management of blood pressure, body weight, and LDL cholesterol should be carried out when developing CVD prevention interventions in adolescents and young adults. Gender differences in CVD risk factors in adolescents and young adults should be emphasized.

## Strengths and limitations of this study

People aged 15–39 years account for about 38% of the global population [11]. It is critical to provide detailed data information on CVD control strategies for health disparities and inequalities in this distinctive population. GBD 2019 allowed us to provide for the first time the most consistent and detailed global distribution and change of CVD disease burden in adolescents and young adults and an overview of its risk factors, with in-depth comparisons of changing trends by sex and age, by country or region group, and by risk factors. However, there were some limitations our study. First, data on CVD in adolescents and young adults in low socioeconomic countries were lacking or of very poor quality, limited by poor screening and data registration. GBD 2019 relied on available epidemiological findings and used several techniques to reduce bias and inaccuracy. Although this cannot completely rule out bias, the criteria for data calculation were consistent and the quality of the data is assured, Second, GBD 2019 lacked the division of the population aged 40 years and older, so we cannot compare the characteristics of CVD burden between adolescents and young adults and adults aged 40 years and older. Third, in calculating age-standardized rates, we used confidence intervals instead of uncertainty intervals, which requires caution in the interpretation of our findings and requires validation with the use of more real-world studies.

## Conclusions

The burden of CVD in adolescents and young adults is a serious health problem globally, especially among males and in countries with low to middle levels of SDI. Special attention should be paid to the prevention of RHD in the younger age group. High systolic blood pressure, high body-mass index and high LDL cholesterol were the main attributable risk factors. Furthermore, attributable risk factors for CVD differed among adolescents and young adults across countries with different SDI levels, genders, and age groups, underscoring the importance of targeted measures to address this growing global health problem.

### Electronic supplementary material

Below is the link to the electronic supplementary material.

Supplementary Material 1

## Data Availability

The datasets used and analyzed during the current study available from the corresponding author on reasonable request.

## Abbreviations

CVD: Cardiovascular disease
GBD 2019: Global Burden of Disease, Injury, and Risk Factor Study 2019
SDI: Sociodemographic index
ASRs: Age-standardized rates
DALYs: Disability-adjusted life years
YLDs: Years lived with disability
YLLs: Years of life lost
ASIR: Age-standardized incidence rate, ASMR: Age-standardized mortality rate
ASDR: Age-standardized DALYs rate. PAFs: Population Attributable Fractions
UI: Uncertainty interval
CI: Confidence interval
EAPCs: Estimated annual percentage changes
