# Supplementary material for: The global burden and risk factors of cardiovascular diseases in adolescent and young adults, 1990–2019
Source: BMC Public Health. 2024 Apr 12;24:1017. doi: 10.1186/s12889-024-18445-6 (PMC11010320; doi:10.1186/s12889-024-18445-6)
Supplement: Supplementary file 1 — Supplementary Material 1 [file 12889_2024_18445_MOESM1_ESM.docx]

**Supplementary Material**

**
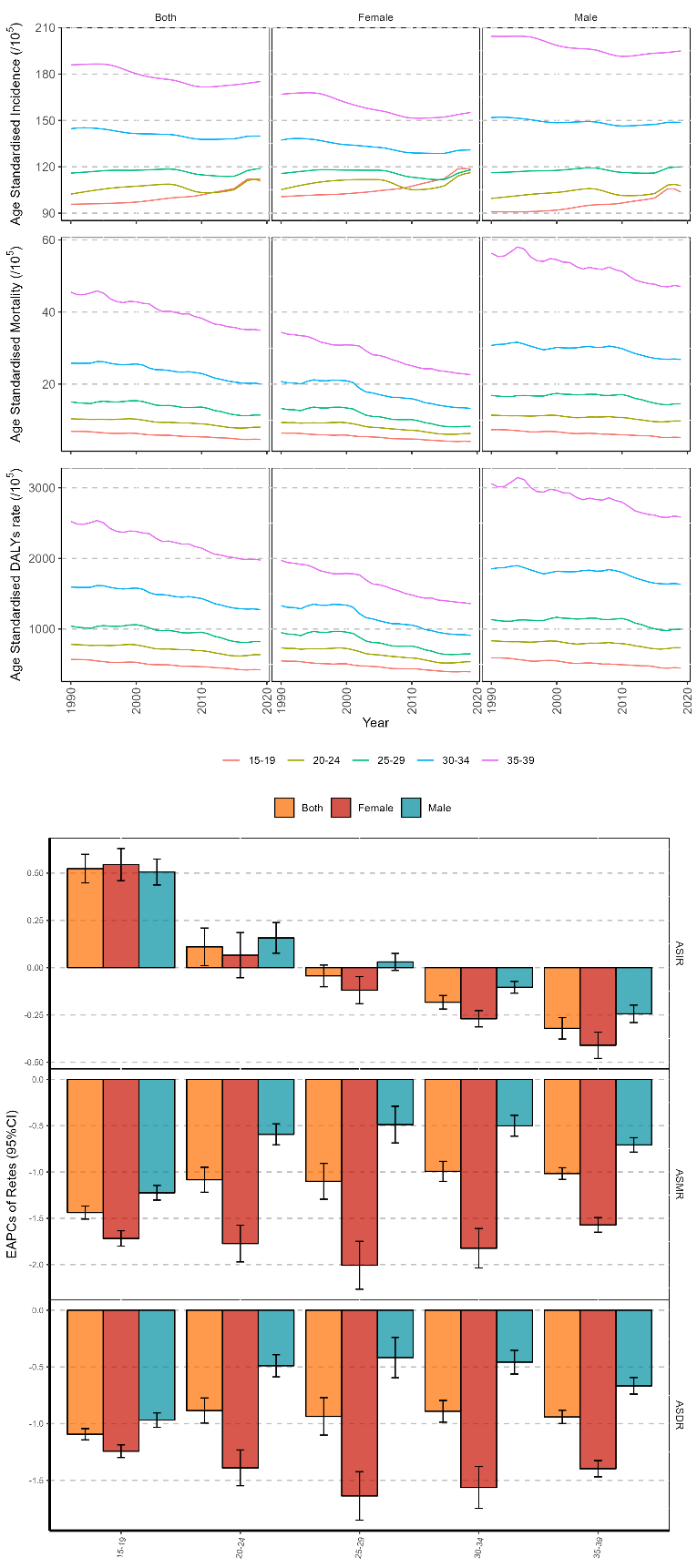
**

Figure S1 Temporal trend of cardiovascular disease (CVD) burden in [adolescent and young adult](http://www.webofscience.com.zzulib.vpn358.com/wos/alldb/full-record/WOS:000821937900038)s by age group from 1990 to 2019.


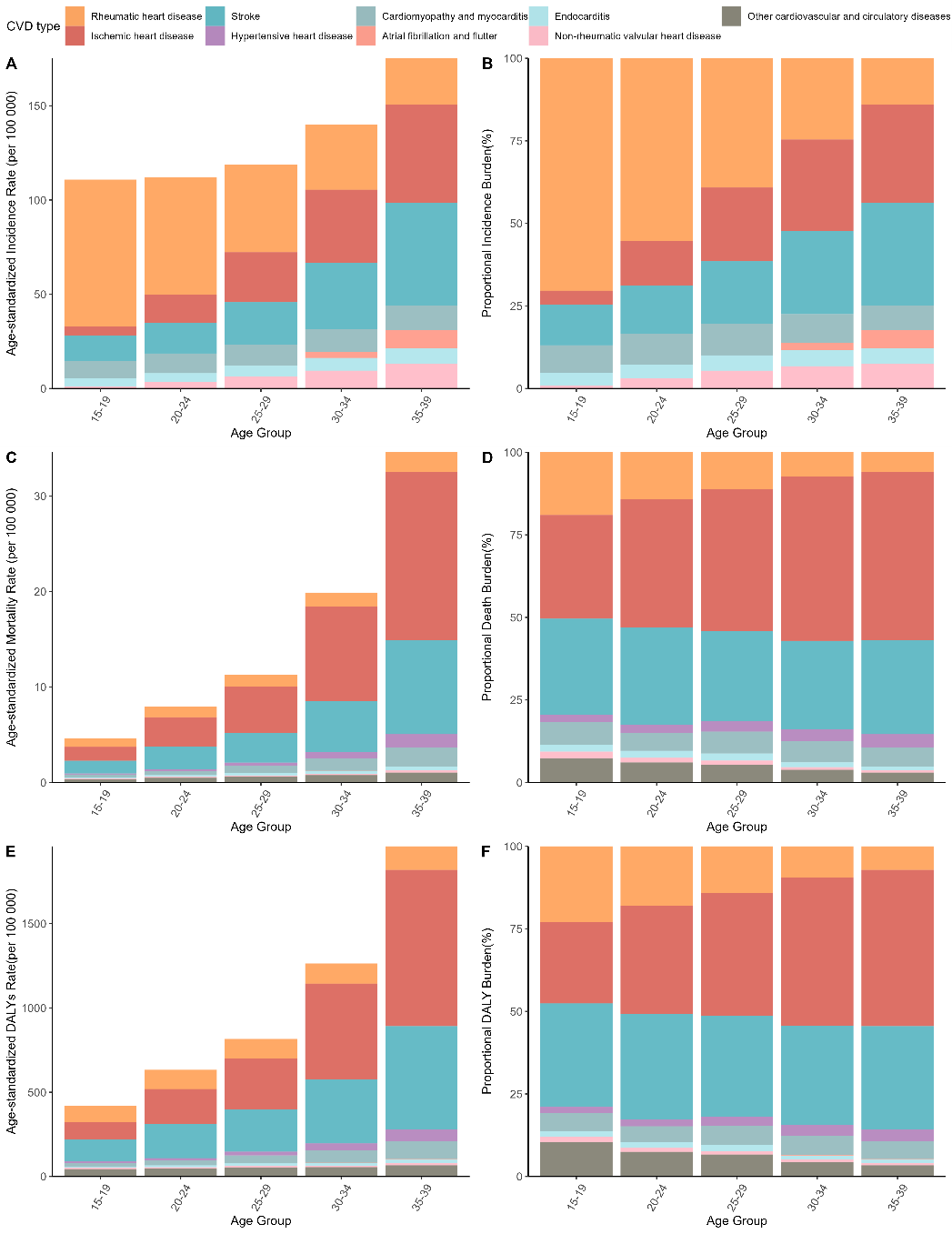


Figure S2 Proportion of Cardiovascular disease (CVD) types globally in [adolescent and young adult](http://www.webofscience.com.zzulib.vpn358.com/wos/alldb/full-record/WOS:000821937900038)s by age group in 2019.


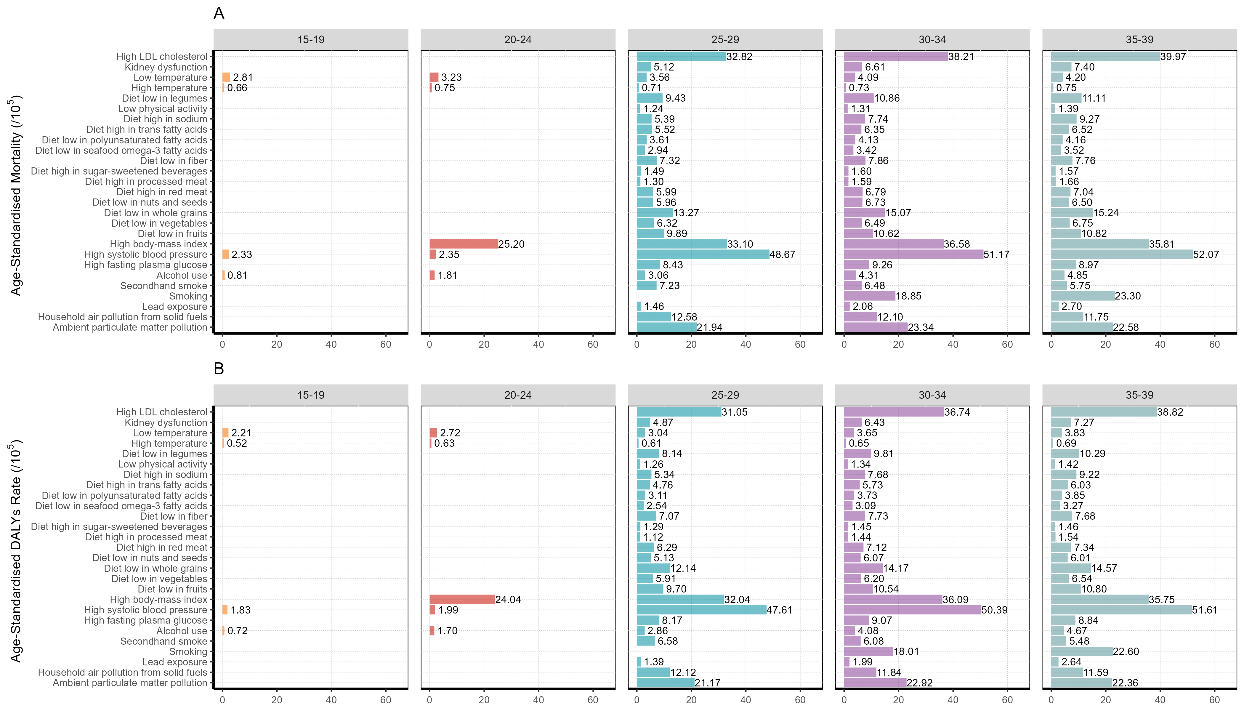


Figure S3 Proportion of Cardiovascular disease (CVD) death and disability adjusted life years (DALYs) in [adolescent and young adult](http://www.webofscience.com.zzulib.vpn358.com/wos/alldb/full-record/WOS:000821937900038)s attributable to 27 risk factors by age group in 2019.
